# “Lessons to Be Learned After the Storm”—A Retrospective Study on the Characteristics and Management of Dental Emergency Patients During the COVID-19 Outbreak in Riyadh

**DOI:** 10.3390/healthcare13050448

**Published:** 2025-02-20

**Authors:** Ali AlAqla, Naif Alrubaig, Kiran Iyer, Adeeb Alshareef, Mohammed Alkathiri, Dana Albassri

**Affiliations:** 1 Department of Restorative and Prosthetic Dental Sciences, College of Dentistry, King Saud bin Abdulaziz University for Health Sciences, Riyadh 11426, Saudi Arabia; aqlaa@ksau-hs.edu.sa (A.A.); alrubaign@ksau-hs.edu.sa (N.A.); alkathirimo@ksau-hs.edu.sa (M.A.); 2 King Abdullah International Medical Research Centre, Ministry of National Guard Health Affairs, Riyadh 11481, Saudi Arabia; 3 Preventive Dental Sciences Department, College of Dentistry, King Saud bin Abdulaziz University for Health Sciences, Riyadh 11426, Saudi Arabia; 4 College of Dentistry, Dar Al Uloom University, Riyadh 13314, Saudi Arabia; adeeb3070@hotmail.com; 5 Department of Training, Ministry of Health, Riyadh 12233, Saudi Arabia; danaalbassri@gmail.com; 6 Department of Endodontics, Dammam Medical Complex, Dammam 32253, Saudi Arabia

**Keywords:** COVID-19, dentistry, oral health, endodontics, antibiotics, analgesics

## Abstract

**Background/Objectives:** There is a limited understanding of the variables relating to dental patients and the treatment provided during the initial phase of the COVID-19 lockdown in our region. This study aimed to qualitatively analyze these patient variables and determine the associations between treatment recommendations and the specialty of the doctor at the point of care. **Methods:** The present study was retrospective, cross-sectional, and analytical in nature. Data regarding symptoms, diagnosis, treatment, and the attending specialist were retrieved from the patient management software for patients seeking emergency dental services during the COVID-19 lockdown (23 March 2020 to 23 April 2020) in primary and tertiary public hospitals of the National Guard Health Affairs in Riyadh, Saudi Arabia. The association between exploratory (symptoms, diagnostic tool, specialist at point of care) and dependent variables (given diagnosis and treatment) was assessed using Fisher’s exact test and multinomial regression analysis. **Results:** A total of 151 dental patients attended the outpatient/emergency clinics during this period. The mean age of the patients in this study was 31.4 (±19.0) years. Compared to physicians, general dentists [OR 0.56, CI 0.29–10.47] were more likely to give an inappropriate diagnosis and treatment, whereas residents [OR 2.70, CI 1.65–98.17] and resident endodontists [OR 2.30, CI 1.28–78.11] were more likely to give an appropriate diagnosis and treatment. **Conclusions:** The findings of this study highlight the need for a greater number of endodontists at the forefront of screening and providing dental care during such health catastrophes.

## 1. Introduction

The world witnessed an alarming number of detrimental pneumonia cases in Wuhan, China, in December 2019, the etiology of which was initially unknown; however, timely intervention by epidemiologists and scientists enabled us to determine that this viral strain was a novel Coronavirus 2 (SARS-CoV-2)/severe acute respiratory syndrome-CoV-2 [1]. Initially, the WHO stated that the disease was a “Public Health Emergency of Internation Concern”. As the virus spread across geographical borders, the World Health Organization (WHO) declared it a pandemic on 11 March 2020 [2].

The first case in Saudi Arabia was detected on 2 March 2020 [3]. Within three weeks, on 23 March 2020, a partial lockdown was put in place, as 562 cases had been reported in the country. Eventually, a complete lockdown was implemented on 6 April 2020, since the number of reported positive cases had increased to 20,605. The lockdown was imposed to curb the spread of infection, which predominantly occurred via the aerial route (aerosols/droplets) [4].

The lockdown consisted of several restrictions, as per the international protocols put in place. The lockdown was implemented at various intensities until 21 May 2022. All institutions (academic, commercial, and cultural) ceased functioning except for those providing medical care, which had emergency and outpatient wards open to treat patients [5]. These operated separately from specialized flu clinics, which handled COVID-19 cases.

In line with the approach taken by most countries, elective dental treatments in the region were postponed [6]. The management of emergency dental cases switched to a less invasive system that involved more screening, minimizing procedures that involved the production of aerosols in order to avoid the transmission of COVID-19 [7]. There were no guidelines prior to the pandemic concerning the approach and management of cases with dental emergencies during such a wide-spread natural catastrophe [4].

The Ministry of Health (MOH), Saudi Arabia, issued a guideline for the management of dental emergency cases in the country in April 2020 [8]. The American Dental Association (ADA)’s infection control protocol and guidelines for patient management during the pandemic were published on 19 May 2020 [9].

Presently, the literature on this topic is limited to questionnaire-based assessments of social determinants during emergency and the treatment that the population received; this includes a study by Meisha D.E et al. [6]. Meanwhile, some studies have analyzed the most common dental symptoms/treatments reported by patients when entering the emergency department during the lockdown phase of the pandemic. There is a lacuna in the literature regarding how the diagnosis was reached, which specialists were available at the point of care, what treatment was administered in most dental emergency cases, and whether the treatment was appropriate [6,10,11]. To our knowledge, no studies in this region have examined the characteristics (age, gender, dental symptom) of dental patients and their subsequent management (radiographs, diagnosis, treatment, and attending specialist to the patients) at outpatient/emergency clinics of major hospitals in Saudi Arabia during the pandemic.

Hence, the aim of this study was to assess all the characteristic variables and the management of dental patients who reported to the dental outpatient/emergency department of one of the largest public hospitals and its tertiary centers during the early phase of the COVID-19 pandemic.

## 2. Methods

The methodology of this study is being reported as per the STROBE guidelines for cross-sectional studies.

### 2.1. Study Design

The present study is retrospective, cross-sectional, and analytical in nature.

### 2.2. Study Setting

The data were retrieved from the hospital-based database (BEST care/SALUD) records of patients who sought emergency dental services during the COVID-19 lockdown in primary and tertiary public hospitals of the National Guard Health Affairs in Riyadh, Saudi Arabia. The data collected were limited to the lockdown period from 23 March to 23 April 2020.

### 2.3. Participants

All the patients who visited the primary and tertiary care centers of the National Guard Hospital for emergency dental care services, as per the patient database records, were included in the study. There were no exclusion criteria set, as all patient-based data were considered for analysis during the aforementioned period.

### 2.4. Exploratory and Dependent Variables

Data were collected at all the emergency care centers through a unified recording platform (patient management software). The data consisted of patient-based demographic inputs such as the patients’ enrollment number, gender, date of birth and age, and whether they reported to the outpatient (OP)/emergency room (ER). Other exploratory (independent) variables were recorded from the software; these included the reporting hospital, the type of diagnostic department, ICD 10 (CODE), the diagnosis, the attending physician/dentist (along with their specialty ranking), the type of record form, the patient’s symptoms, and the X-ray/diagnostic aid suggestion.

The diagnosis and treatment received by patients were considered as dependent variables in the analysis.

### 2.5. Bias and Study Size

Since the present study is a retrospective secondary analysis of a patient database, it is possible that observation bias was inherent in the data collection.

The study included all patients included in the database during the aforementioned period; hence, sample size estimation was not carried out in the present study.

### 2.6. Statistical Analysis

The data were entered into Microsoft Excel and transferred to Statistical Package for Social Sciences (SPSS) statistical software version 20 (IBM Corp, Chicago, IL, USA) for analysis. Descriptive statistics were used to describe all the qualitative variables and demographic data. Pearson’s chi-square analysis was used to determine the significance of patient management among the specialist doctors available at the point of care, along with the exploratory variables. Significant variables were further analyzed using multinomial regression analysis.

## 3. Results

The dental records of 151 patients were assessed using the common database adopted across various National Guard Health Affairs hospital centers. These records, which include dental patients reporting to the emergency and outpatient departments of these centers, were assessed during the complete lockdown, namely between 23 March 2020 and 23 April 2020.

The mean age of patients in the study was 31.4 (±19.0) years. The minimum and maximum ages of the patients were 2 years and 76 years, respectively. During the period considered for observation, 85 males (56.3%) reported to the outpatient department, compared to 66 females (43.7%). Most of the patients, namely 81 (53.6%), with oral health-related complaints during this period reported to the emergency department; a high number of these patients, namely 113 (74.8%), reported to the Hospital of King Abdullah Medical City-Riyadh (KAMC-R) (Table 1).

While pain (27.2%) and swelling (25.8%) were the most symptoms most commonly reported by the patients, data about the symptoms of 44 (29.1%) patients were unavailable and were hence categorized under no data. In accordance with the most common symptoms reported, the clinical diagnosis of periapical abscess (40.4%) and pulpitis (23.2%) was evident in the data. Antibiotics were prescribed to 59 (39.1%) of the patients (Table 2).

Fisher’s exact test was used to determine whether significance existed between the variables. When symptoms were analyzed in relation to the performance of a pre-operative X-ray, most intra-oral periapical (IOPA) radiographs, namely 13 (8.6%), were taken when the patient complained of pain; meanwhile, orthopantomography (OPG) was considered in three (2.0%) cases of swelling. On the whole, radiographs were not taken in 83 (55.0%) of the patients; however, 38 (25.2%) of the patients were recommended for pre-operative radiographs (*p* < 0.00) (Table 3).

Similarly, significance (*p* < 0.00) was observed with Fisher’s exact test when treatment was assessed based on symptoms. The majority of the patients were dismissed with medication, antibiotics were prescribed for 59 (39.1%) patients, and analgesics were prescribed for four (2.6%) patients; therefore, a total of 63 (41.7%) patients were treated using medication only. Treatment was undertaken in a total of 39 (22.6%) patients; this consisted of 14 (9.3%) endodontic treatments, 9 (6.0%) extractions and approximately 11 (7.3%) treatments that were included in the “other” category (oral hygiene procedures, mouthwash recommendations, etc.) (Table 3).

Crosstabulation was also used to analyze the patients’ diagnosis based on the specialty of the doctor at the frontline during the period. A significant (*p* < 0.00) associated between the specialty of the doctor and the diagnosis was observed, with periapical abscess and pulpitis mostly being diagnosed by physicians and dentists (Table 3).

A final crosstabulation was carried out to compare the medication (analgesic/antibiotic) prescribed and the endodontic treatment/referral suggested, based on the patient’s symptoms, with the specialty of the doctor at the point of contact. There was a significant difference (*p* = 0.01) observed, as physicians prescribed antibiotics for pain and swelling more frequently than the other specialists (Table 4).

Multinomial logistic regression analysis was carried out to determine whether there were discrepancies in the diagnosis and treatment administered between various specialty doctors. Because the majority of the cases had pulpal involvement, a board-certified endodontist was asked to categorize the diagnosis and treatment into appropriate and inappropriate based on the patients’ pre-operative X-ray findings, as well as the diagnosis and treatment considered by the specialty doctors.

The analysis revealed a significant difference in the way the various specialty doctors diagnosed and suggested/carried out the treatment. Compared to physicians, general dentists [OR 0.56, CI 0.29–10.47] were similar in their provision of an inappropriate diagnosis and treatment, whereas residents [OR 2.70, CI 1.65–98.17] and resident endodontists [OR 2.30, CI 1.28–78.11] were more likely to provide appropriate diagnosis and treatment (Table 5).

## 4. Discussion

This study provides crucial data regarding dental care utilization patterns and practitioners’ availability at the point of care. It determines the type of diagnosis, treatment, and referral made by practitioners during the initial phase of the COVID-19 pandemic lockdown. This phase of the pandemic was marred by uncertainty for doctors in emergency and outpatient services, who were on the frontlines of duty [12,13].

As most routine dental care was unavailable during the pandemic, patients were expected to seek emergency dental services when needed. Moreover, there were no previous guidelines regarding what would qualify as emergency dental care [4]. Tempro-mandibular joint disorders (TMDs) and bruxism, which are direct outcomes of psychological and emotional stress, were not considered to be emergency dental care in most countries during the pandemic; however, the MOH, which was Saudi Arabia’s guideline on dental emergencies during the COVID-19 pandemic, did consider temporomandibular arthralgia/myalgia to be urgent care and provided physicians with guidance on its management [7,14].

This study reports findings related to emergency dental care in patients reporting to a major public hospital and its tertiary care centers in Riyadh, Saudi Arabia, from the onset of the COVID-19 lockdown. Anxiety gripped society during this phase of the pandemic; hence, outpatient department saw a drastic decrease in patient flow. A similar finding was reported in China, where the overall number of patients reporting to the emergency dental department was reduced by 38% due to lockdown restrictions and a fear of venturing out [11].

A literature search revealed that a similar study was performed in a large public hospital (AlJahra) in Kuwait (a neighboring country to Saudi Arabia); the data were based on a sample of 34,250 patients, and the time frame covered two years and included pre- and post-pandemic patients who were assessed for treatment patterns [15]. The study reported a drop in patients seeking dental care and dental procedures during the initial lockdown phase; this started to return to normal, but did not reach pre-COVID-19 levels. In non-regional research, a similar finding was observed in Poland; based on a questionnaire study, it was found that more than half of the respondents feared accessing dental care [16]. A high number of male patients reported to the emergency dental department during the pandemic at the hospitals considered in the study; this finding is similar to those reported in other studies from the region, namely those by Olayan, A.A et al. [17] and Yaqin Syed, A.U et al. [18]. In this study, pain was the most common dental symptom reported by the patients in the emergency department, similar to studies by Sun, Q et al. [19] and Moca, A.E et al. [20], respectively.

A significant finding of this study was the presence of physicians at the point of care for dental emergencies, which does reflect the low percentage of dental radiographs (IOPA/OPG) taken during this period. This may be attributed to concerns related to saliva contamination among the physicians, the X-ray facilities being integrated into open clinics, a lack of radiology technicians, or a lack of awareness regarding the need for radiographs in specific dental emergencies. Studies do suggest that radiology units have a lower likelihood of facilitating disease transmission, either through direct or indirect methods, than other specialties in dentistry [21].

Moreover, the viral load of asymptomatic patients was similar to that of symptomatic patients, and approximately 20% to 60% of patients had minor nonspecific symptoms [22]. In such a scenario, it is difficult to determine which patients could and could not be provided with dental services; hence, all patients were treated with a uniform policy.

International guidelines for dental practice during the pandemic came into existence only one the nature of the virus was known; until then, there were no guidelines for dental practice in epidemic or pandemic situations. The American Dental Association (ADA) mandated that all dental clinics stop functioning until 30 April 2020 [9]. The earliest guidelines for infection control during the COVID-19 pandemic in dental practice were provided on 19 May 2020. Interestingly, a paper presenting guidelines for emergency dental care in Saudi Arabia was published early on in the pandemic (by the first week of April 2020) [4]. The Ministry of Health (MOH) only established a protocol for the treatment of dental patients in the second week of April 2020 [8]. Subsequently (9 months after the COVID-19 pandemic began), a study by Basheer SN et al. [23] reflected upon the low levels of knowledge that dental healthcare personnel had about the COVID-19 protocol published by the MOH.

In the present study, it is clear that dental specialists are needed at the frontline for screening, diagnosis, and treatment at emergency dental clinics; endodontists are particularly important, considering that most dental symptoms and the final diagnosis are related to odontogenic infections [24,25]. The high percentage of people with periapical abscesses and the rate at which these patients were discharged with antibiotics for pain are appalling. This corroborates with findings presented by the American Association of Endodontists (AAE) [26].

In a systematic review conducted by Lockhart PB et al., under the aegis of the American Dental Association Council and Center for Evidence-based Dentistry, clinical recommendations for the emergency management of symptomatic irreversible pulpitis with or without periapical infection were formulated; these state that there is no need for the prescription of antibiotics unless the patient exhibits systemic involvement, such as fever/malaise arising from dental infection. These recommendations were primarily directed at general dentists, but they could be used by dental specialists, public health dentists, emergency and primary care physicians, and policymakers. These recommendations can be deliberated upon and help with the development of policy frameworks [27].

The present study concludes that endodontist/endodontic residents play a highly significant role in ensuring that the appropriate diagnosis and treatment are given compared to physicians and general dentists. Chaudhary FA’s systematic review on endodontic treatment during the COVID-19 pandemic resonates with this conclusion [28]. In the future, patient-based software that is trained using artificial intelligence datasets for dental diagnosis and treatment could be used; this is in order to reduce the occurrence of observational bias when various specialty doctors are involved [29].

## 5. Conclusions

COVID-19 has affected the world in many ways, particularly as the last pandemic known to humankind occurred more than a century ago; the present study provides crucial insights into the region’s preparedness to handle dental emergencies during the initial lockdown of the COVID-19 pandemic. The present study suggests the need to train frontline physicians in essential dental diagnosis and referral, in addition to the appropriate medication guidelines; this is especially important regarding the prescription of antibiotics for patients reporting dental pain, unless there is a systemic indication. Furthermore, a clear policy regarding dental radiography is needed; this is in order to ensure that physicians are aware of the minimal risk of cross contamination it poses. The internal policy that exists for the diagnosis and management of dental patients was not analyzed, and this is one of the main limitations of our study. The suggestions are based on findings from patients reporting to a single public hospital and its tertiary centers, which is why the sample size is limited. In addition, the study does not provide deep insights into findings related to patients and the management of dental emergencies; hence, the results and conclusions should be interpreted with caution. While the sample size was small, our findings are still generalizable, as is evident in the comparisons we draw with similar studies (early in the COVID-19 pandemic).

Endodontists, endodontic residents, and dental residents played a key role in managing dental emergency patients, suggesting that their posting and availability is crucial for the appropriate care of dental patients during pandemics or epidemics.

## Figures and Tables

**Table 1:** Demographics of the patients attending the dental outpatient department.

	Demographic Variable	Total (N)	Mean	Standard Deviation (±)	Frequency (n)	Percentage (%)
Age of Patients (Years)	Male	151	32.9	20.1	85	56.3
	Female		29.3	17.5	66	43.7
			31.4	19.0		
Patient Type	Emergency (ER)	151	-	-	81	53.6
	Outpatient (OP)				70	46.4
Gender	Male	151	-	-	85	56.3
	Female				66	43.7
Hospital Visited	DIRAB (Area)	151	-	-	09	6.0
	ISKAN (Area)				13	8.6
	King Abdullah Medical City-Riyadh (KAMC-R)				113	74.8
	King Khalid Medical City (KKMC)				03	2.0
	Ministry of National Guard Clinics (MNGC)				02	1.3
	National Guard Comprehensive Specialized Clinics (NGCSC)				11	7.3
Symptoms	Pain	151	-	-	41	27.2
	Swelling				39	25.8
	Abscess				19	12.6
	Other Symptoms *				08	5.3
	No Data				44	29.1

* Other Symptoms—fractured tooth (1), chemotherapy patient (1), bleeding gums (6).

**Table 2:** Frequency and percentage of various diagnoses and treatments, as observed in the data.

	Category	Total	Frequency	Percentage
Diagnosis	Periapical abscess	151	61	40.4
	Dental caries		34	22.5
	Gingivitis		14	9.3
	Oral ulcer		04	2.6
	Periodontitis		03	2.0
	Pulpitis		35	23.2
Treatment	Analgesic		04	2.6
	Antibiotic		59	39.1
	Extraction		09	6.0
	Endodontic treatment		14	9.3
	Referred		07	4.6
	No data		27	17.9
	No treatment or medication		20	13.2
	Other		11	7.3

**Table 3:** Crosstabulation to assess the significance between the independent (symptoms and diagnosis) variables and dependent variables (X-ray/treatment/specialist).

Symptoms	Pre-operative X-ray													
	Yes		OPG		NO IOPA/OPG		Wrong MRN		No Information		Total		df	Fisher’s Exact Test (Sig)
Pain	13 (8.6)		0 (0.0)		24 (15.9)		0 (0.0)		4 (2.6)		41 (27.2)		16	*p* = 0.00 *
Abscess	6 (4.0)		0 (0.0)		13 (8.6)		0 (0.0)		0 (0.0)		19 (12.6)			
Swelling	5 (3.3)		3 (2.0)		30 (19.9)		0 (0.0)		1 (0.7)		39 (25.8)			
No data	11 (7.3)		0 (0.0)		12 (7.9)		4 (2.6)		17 (11.3)		44 (29.1)			
Other symptoms	3 (2.0)		0 (0.0)		4 (2.6)		0 (0.0)		1 (0.7)		8 (5.3)			
Total	38 (25.2)		3 (2.0)		83 (55.0)		4 (2.6)		23 (15.2)		151 (100)			
Symptoms	Treatment													
	Analgesic	Antibiotic		Extraction	Endodontic Treatment	Referred		No Information	No Treatment or Medication	Other		Total	df	
Pain	3 (2.0)	18 (11.9)		3 (2.0)	9 (6.0)	1 (0.7)		2 (1.3)	3 (2.0)	2 (1.3)		41 (27.2)	28	*p* = 0.00 *
Swelling	1 (0.7)	24 (15.9)		3 (2.0)	0 (0.0)	0 (0.0)		2 (1.3)	6 (4.0)	3 (2.0)		39 (25.8)		
Abscess	0 (0.0)	9 (6.0)		0 (0.0)	2 (1.3)	0 (0.0		1 0.7	5 3.3	2 1.3		19 12.6		
No data	0 (0.0)	5 (3.3)		3 (2.0)	3 (2.0)	4 (2.6)		22 (14.6)	4 (2.6)	3 (2.0)		44 (29.1)		
Other symptoms ^#^	0 (0.0)	3 (2.0%)		0 (0.0)	0 (0.0)	2 (1.3)		0 (0.0	2 (1.3)	1 (0.7)		8 (5.3)		
Total	4 (2.6)	59 (39.1)		9 (6.0)	14 (9.3)	7 (4.6)		27 (17.9)	20 (13.2)	11 (7.3)		151 (100)		
Diagnosis	Speciality													
	Dentist			Endodontist		Physician			Residents			Total	df	Fisher’s Exact Test (Sig)
Periapical abscess	20 (13.2)			1 (0.7)		30 (19.9)			7 (4.6)			58 (38.4)	15	*p* = 0.00 *
Dental caries	13 (8.6)			4 (2.6)		11 (7.3)			7 (4.6)			35 (23.2)		
Gingivitis	7 (4.6)			0 (0.0)		6 (4.0)			0 (0.0)			13 (8.6)		
Pulpitis	11 (7.3)			0 (0.0)		23 (15.2)			3 (2.0)			37 (24.5)		
Chronic apical periodontitis	0 (0.0)			3 (2.0)		0 (0.0)			0 (0.0)			3 (2.0)		
Other	2 (1.3)			0 (0.0)		2 (1.3)			1 (0.7)			5 (3.3)		
Total	53 (35.1)			8 (5.3)		72 (47.7)			18 (11.9)			151 (100)		

* Significance = (*p* < 0.05); other symptoms ^#^ fractured tooth (1), chemotherapy patient (1), bleeding gums (6).

**Table 4:** Medication and endodontic treatment/referral for symptoms (pain, swelling, and abscess) based on the specialist at the point of contact.

Symptoms	Treatment	Ranking/Specialty				Total	df	Fisher’s Exact Test
		Dentist	Endodontist	Physician	Residents			
Pain	Analgesic	1 (2.4)	0 (0)	1 (2.4)	1 (2.4)	3 (7.3)	15	0.01 *
	Antibiotic	6 (14.6)	1 (2.4)	9 (22.0)	2 (4.9)	18 (43.9)		
	Endodontic Treatment/Referral	4 (9.8)	2 (4.9)	2 (4.9)	1 (2.4)	9 (22.0)		
	Total	11 (26.4)	3 (7.2)	12 (28.8)	4 (9.6)	30 (72.0)		
Swelling	Analgesic	0 (0)	0 (0)	1 (2.6)	0 (0)	1 (2.6)		
	Antibiotic	4 (10.3)	2 (5.1)	14 (35.9)	4 (10.3)	24 (61.5)		
	Extraction	2 (5.1)	0 (0)	0 (0)	1 (2.6)	3 (7.7)		
	Total	6 (28.8)	2 (5.1)	15 (39.0)	5 (13.0)	28 (72.8)		
Abscess	Analgesic	1 (5.3)	NA	1 (25.0)	1 (25.0)	3 (75.0)		
	Antibiotic	2 (10.5)	NA	6 (31.6)	1 (5.3)	9 (47.4)		
	Endodontic Treatment/Referral	1 (5.3)	NA	0 (0)	1 (5.3)	2 (10.5)		
	Total	3 (15.9)	NA	7 (37.1)	3 (15.9)	14 (74.3)		

* Significance = (*p* < 0.05).

**Table 5:** Multinomial regression used to assess significance of diagnosis and treatment based on specialty.

Va riable	Independent Variable	B	df	Sig.	Exp (B)	95% Confidence Interval for Exp (B)	
						Lower Bound	Upper Bound
Endodontist Expert Opinion on Appropriate Diagnosis and Treatment	General Dentist	0.56	1	0.43	1.76	0.29	10.47
	Resident	2.70	1	0.01 *	15.0	1.65	98.17
	Resident Endodontist	2.30	1	0.02 *	10.0	1.28	78.11
	Physician	0 ^#^	-	-	-	-	-

^#^—Reference category; last category was considered the reference; B—odds; and * significance = (*p* < 0.05).

## Data Availability

Data are available in a publicly accessible repository. The data presented in the study are openly available in [Fig Share] at 10.6084/m9.figshare.28052555 (accessed on 18 December 2024).
